# Sexual Preferences and Presentation on Geosocial Networking Apps by Indian Men Who Have Sex With Men in Maharashtra

**DOI:** 10.2196/mhealth.5600

**Published:** 2016-10-31

**Authors:** Jayson Rhoton, J Michael Wilkerson, Shruta Mengle, Pallav Patankar, BR Simon Rosser, Maria L Ekstrand

**Affiliations:** ^1^ Department of Health Promotion and Behavioral Sciences The University of Texas Health Sceince Center Houston Houston, TX United States; ^2^ The Humsafar Trust Mumbai, Maharashtra India; ^3^ School of Public Health University of Minnesota Minneapolis, MN United States; ^4^ Center for AIDS Prevention Studies University of California San Francisco San Francisco, CA United States

**Keywords:** homosexuality, mobile health, HIV, prevention and control, urban health

## Abstract

**Background:**

The affordability of smartphones and improved mobile networks globally has increased the popularity of geosocial networking (GSN) apps (eg, Grindr, Scruff, Planetromeo) as a method for men who have sex with men (MSM) to seek causal sex partners and engage with the queer community. As mobile penetration continues to grow in India, it is important to understand how self-presentation on GSN app is relevant because it offers insight into a population that has not been largely studied. There is very little information about how Indian MSM discuss their sexual preferences and condom preferences and disclose their human immunodeficiency virus (HIV) status with potential sex partners on Web-based platforms.

**Objective:**

The objective of this study was to describe how self-presentation by Indian MSM on GSN apps contributes to sexual preferences, HIV or sexually transmitted infection (STI) disclosure, and if the presentation differs due to proximity to the Greater Mumbai or Thane region.

**Methods:**

Between September 2013 and May 2014, participants were recruited through banner advertisements on gay websites, social media advertisements and posts, and distribution of print materials at outreach events hosted by lesbian, gay, bisexual, transgender (LGBT) and HIV service organizations in Maharashtra, India. Eligible participants self-identified as being MSM or hijra (transgender) women, living in Maharashtra, aged above 18 years, having regular Internet access, and having at least one male sex partner in the previous 90 days.

**Results:**

Indian MSM living inside and outside the Greater Mumbai or Thane region reported an average of 6.7 (SD 11.8) male sex partners in the last 3 months; on average HIV status of the sex partners was disclosed to 2.9 (SD 8.9). The most commonly used websites and GSN apps by MSM living inside Greater Mumbai or Thane region were Planetromeo, Grindr, and Gaydar. Results demonstrated that MSM used smartphones to access GSN apps and stated a preference for both condomless and protected anal sex but did not disclose their HIV status. This low level of HIV disclosure potentially increases risk of HIV or STI transmission; therefore, trends in use should be monitored.

**Conclusions:**

Our data helps to fill the gap in understanding how Indian MSM use technology to find casual sex partners, disclose their sexual preference, and their HIV status on Web-based platforms. As mobile penetration in India continues to grow and smartphone use increases, the use of GSN sex-seeking apps by MSM should also increase, potentially increasing the risk of HIV or STI transmission within the app’s closed sexual networks.

## Introduction

The affordability of smartphones and improved mobile networks globally has likely contributed to the increased popularity of geosocial networking (GSN) apps (eg, Grindr, Scruff, and Planetromeo) as a method for men who have sex with men (MSM) to seek causal sex partners and engage with the queer community [1-3]. The growth in popularity of GSN apps has been attributed to the global positioning systems and algorithms that match men based on shared interests, sexual attraction, muscularity, age, and proximity [4,5]. In Western MSM populations, GSN apps have shown to be more popular than other online peer-to-peer social networking sites (eg, Facebook) and traditional methods (eg, cursing area and gay bars) to find causal sex partners [4-6]. Several studies have found that MSM report logging in to GSN apps at least three times a day, with an average of 12 minutes being spent per log-in [3,7-9].

Till date, research on MSM’s use of GSN apps has focused on urban and rural Western MSM populations, examining sexual risk behaviors, partner preferences, condom use, and human immunodeficiency virus (HIV) or sexually transmitted infection (STI) disclosure [3]. Western MSM who use GSN apps to find causal sex partners who tend to be younger and have more sexual encounters, which potentially increases their risk of HIV or STI infection [5,7,10]. While the evidence is unclear if GSN apps actually contribute to increase in sexual risk behavior [4,7], several studies have reported that MSM who used GSN apps had higher lifetime male anal sexual partners and reported greater condomless anal-receptive sex, condomless oral-sex, and rimming, than MSM who used offline venues [11-14]. Additionally, MSM using GSN apps report to have on average more sexual partners, greater encounters with partners known to have HIV and STI diagnoses [4,11], than MSM meeting sex partners offline.

The use of GSN apps as a tool for seeking sexual partners raises the questions of how urban and rural MSM present themselves on Web-based platforms and how they disclose their sexual and condom preferences and HIV or STI status [3,15]. Suler and others exploration of self-presentation on GSN and social networking apps for MSM has found that MSM Web profiles offer dissociative anonymity, which offers MSM the ability to adjust their nonvirtual identities based on their sexual preference (eg, topping, bottoming, versatile, condomless sex and protected sex), which have been found to vary across multiple GSN apps [3]. These findings suggest that MSM are creating multiple profiles on GSN apps with different biographical information, sexual position preferences, condom use preferences, and HIV or STI disclosure.

To our knowledge, there is no published data on how Indian MSM use GSN apps to seek sex partners. It is particularly important to understand this gap in the literature as the Telecom Regulatory Authority of India reports that India has 979 million wireless phone users [1]. As Internet and mobile penetration increases in India [2], it is anticipated that the number of MSM using GSN apps to identify male sex partners will increase, similar to Western MSM [11].

As a first step to understanding to what extent the use of GSN apps by Indian MSM mirror the behavioral risks contributing to HIV or STI transmission among Western MSM, descriptive data is needed about which apps Indian MSM use and how they construct an online identity when using these apps. Presentation of self and disclosure of HIV or STI status is important in this context because virtual communities, such as the sexual communities created by GSN apps, provide a space where individuals can explore the boundaries of their sexual attraction and potentially identify persons willing to engage in casual sex. This has been supported in the literature when looking at unidentified gay men, who create multiple profiles on GSN apps to have same-sex encounters [15,16]. Additionally, it is important to understand if there is a difference in sexual preference and HIV disclosure on GSN apps between men living inside or outside the Greater Mumbai or Thane region. We believe that this is an important distinction to make, as men living inside the Greater Mumbai or Thane region are more likely to be exposed to more HIV prevention messaging than men living outside the region. Therefore, the aim of the study was to describe how Indian MSM self-presentation on GSN apps contributes to sexual preferences, HIV or STI disclosure, and if the presentation differs due to proximity to the Greater Mumbai or Thane region.

## Methods

### Recruitment

The recruitment procedure is described in detail elsewhere [17]. Broadly, between September 2013 and May 2014, participants were recruited through banner advertisements on gay websites, social media advertisements and posts, and distribution of print materials at outreach events hosted by LGBT and HIV services organizations in Maharashtra, India. Most participants were recruited from the Greater Mumbai or Thane region. This region has a population of approximately 20 million and is the headquarters for many of India’s largest corporations, the National Stock Exchange of India, and Bollywood. It attracts migrants from other areas of India seeking employment opportunities and an urban lifestyle. Recruitment materials directed participants to the study website where they could complete the eligibility screen. Eligible participants self-identified as being MSM or hijra or transgender woman, living in Maharashtra, above 18 years, having regular Internet access, and having at least one male sex partner in the previous 90 days. Once eligibility was determined, participants were provided with an overview of the study. After completing an online consent procedure [10], an online cross-sectional survey was administered, which took approximately 30 minutes to complete. Participants were able to complete the eligibility screener, consent process, and survey in Hindi, Marathi, or English. Participants were compensated 300 rupees (approximately 4 USD) for completion of the survey. Of the 6049 individuals who clicked on the survey link from an online advertisement, 745 completed consent, 617 initiated the survey, 477 completed the survey, and 449 were eligible. The institutional review boards of the authors’ institutions approved study procedures.

### Measures

#### Use of Geosocial Networking Apps and Self-Presentation

Participants were asked about which websites or GSN apps they used to meet sex partners, the number of profiles on each (eg, Facebook, Desicrossdressers, Planetromeo, Scruff, Grindr, Manjam, and Gaydar), and if they disclosed their preferred sexual position and HIV status on their profile; participants were provided a write-in option. To identify all possible websites or GSN apps, participants were provided with a list that was generated by the authors who work for a nongovernmental organization serving MSM in the Greater Mumbai or Thane region; a write-in option was provided, so that websites and apps not included in the provided list could be captured. To minimize data-entry errors by participants, an algorithm was employed that reminded participants how many profiles they identified; as participants’ wrotein responses, the algorithm subtracted the responses from the total number of profiles identified.

#### Proximity to the Greater Mumbai or Thane Region

Participants were asked in which district of Maharashtra they lived in. Persons who indicated living in Greater Mumbai or Thane were placed in one category and all other participants were placed in another.

#### Demographic Characteristics and Self-Reported Questionnaire

Participants were asked to identify their gender, age, educational degrees obtained, employment status, sexual orientation, openness (outness) about their same-sex attraction, HIV status, and number of female, hijra (transgender) women and male sex partners they had in the last 3 months. Participants self-reported how they stated a preference for condoms and sexual position of their GSN apps.

### Statistical Analysis

Bivariate analyses were used to identify which websites and GSN apps Indian MSM access and the frequency of disclosure of their sexual preferences and HIV status. Chi-square tests (χ^2^) of independence were used to examine the relation between MSM living within or outside of the Greater Mumbai or Thane region, demographic characteristics, Internet-enabled device, and social media platforms. Chi-square tests of independence were also used to examine the differences between disclosure of sexual preferences and HIV status on websites and GSN apps. Fisher’s exact tests were used to identify differences when counts were small.

## Results

A total of 449 MSM completed the survey; 96% (433/449) identified as cisgender males (congruency between gender assigned at birth and current gender identity; this term is used to refer to nontransgender persons). Most participants had completed college and were employed. Over three-quarters of participants self-identified as being gay or bisexual, but only 19% (86/445) reported being open about their same-sex attraction (out) to most or everyone in their life. Participants inside and outside the Greater Mumbai or Thane region reported an average of 6.7 male sex partners (SD 11.8) in the last 3 months; on average HIV status was disclosed to 2.9 (SD 8.9) of the sex partners. Participant’s demographics are presented in Table 1 and Table 2.

**Table 1:** Demographic characteristics of participants (mean, SD; N=449).

Demographics		Mean (SD)
Age in years		29.46 (8.20)
Male sexual partners in the previous 90 days		6.77 (11.80)
**Hours using the Internet**		
	Work-related	22.05 (22.60)
	Searching for sex	7.99 (11.60)
	Meeting potential sex partners	2.36 (5.10)
	Looking at pornography	3.74 (6.70)
	Other activities not related to work, sex, or education	10.27 (13.90)
HIV^a^ status on GSN^b^ app		2.98 (8.96)

^a^HIV: human immunodeficiency virus.

^b^GSN: geosocial networking.

**Table 2:** Demographic characteristics of participants (% values; N=449).

Demographics		n (%)
**Live in Greater Mumbai or Thane region**	
	Yes	335 (74.6)
	No	114 (25.3)
**Earn Rs 25,001 or more**		
	Yes	233 (55.7)
	No	185 (44.2)
**Completed college**		
	Yes	57 (12)
	No	384 (87.0)
**Employment status**		
	Not employed	47 (10)
	Student-not employed	63 (14)
	Student-employed full or part time	33 (7)
	Employed part-time	31 (7)
	Employed full-time	267 (60.5)
**Outness or openness**		
	Out to none	110 (24.7)
	Out to few to half	249 (55.9)
	Out to most to all	86 (19)
**HIV status**		
	Positive	10 (2)
	Negative	336 (75.6)
	Status unknown	98 (22)
**Sexual orientation^a^**		
	Gay or homosexual	2 (0)
	Bisexual	260 (63.7)
	Straight or heterosexual	102 (25.0)
	Double decker or versatile	8 (2)
	Kothi	16 (3)
	Panthi	5 (1)
	Hijra or transgender	3 (0)
	Queer	4 (1)
	Other	8 (1)

^a^In addition to identifying with sexual orientations commonly used in the West, some Indian men who have sex with men identify with sexual orientations specific to the Indian context and frequently associated with a preferred sexual position, including double decker or versatile (someone who typically engages in receptive and insertive sex), kothi (someone who typically engages in receptive sex), and panthi (someone who typically engages in insertive sex).

**Table 3:** Number of sex-seeking profiles used to meet men in the past 12 months by place of residence, September 9, 2013 through June 30, 2014 (N=449).

Sex-seeking platforms	Within the Greater Mumbai or Thane region (N=332)			Outside the Greater Mumbai or Thane region (N=114)			χ^2^_2_	*P* value
Variable	No profiles (n)	1 profile (n)	2 or more profiles (n)	No profiles (n)	1 profile (n)	2 or more profiles (n)		
Planetromeo	68	196	71	12	67	35	7.8	.02
Scruf	293	38	4	105	6	3	4.5	.1
Grindr	201	118	16	87	23	4	9.9	<.001
Desicrossdressers	322	10	3	110	1	3	3.4	.18
Manjam	263	62	10	92	16	6	2.2	.32
Gaydar	287	45	3	102	8	4	6.8	.03
Facebook	166	130	39	58	39	17	1.2	.54

Indian MSM in our sample had GSN apps running in the background for much of the day, reporting an average of 8 hours per day looking for potential sex partners. The most commonly used Internet-enabled devices for sex-seeking were smartphones (292/442, 66%), personal desktop (154/443, 65%), and laptop computers (272/441, 62%). The most commonly used websites and GSN apps by MSM living inside Greater Mumbai or Thane region were Planetromeo, Grindr, and Gaydar. Additionally, MSM living in the Greater Mumbai or Thane region had created more profiles on GSN apps and websites than MSM living outside the Greater Mumbai or Thane region. Despite the difference in websites and GSN apps between groups, a majority of participants had multiple profiles on websites and GSN app (see Table 3).

MSM living within Greater Mumbai or Thane region stated a greater preference for protected insertive and receptive anal sex (SD 6.35, *P*<.001 and SD 5.34, *P*=.02, respectively) on GSN apps and websites compared with MSM living outside Mumbai or Thane region. MSM living in Greater Mumbai or Thane region also showed significant difference in preference for more condomless insertive (SD 5.61, *P*=.01), condomless receptive (SD 4.38, *P*=.04) and condomless versatile anal sex (SD 4.88, *P*=.03) than MSM living outside Greater Mumbai or Thane region. There were no differences between the MSM within and outside of Greater Mumbai or Thane region in stated preference for versatile anal sex with condoms (SD 2.72, *P*=.09) and disclosing their HIV status on websites or GSN apps (SD 0.86, *P*=.65).

## Discussion

### Principal Findings

The purpose of this study was to describe how Indian MSM present themselves on GSN apps, how their presentation contributes to sexual preferences, and if the presentation differs due to proximity to the Mumbai or Thane region. Indian MSM are using smartphone (eg, BlackBerry Bold Touch) as their primary means to access sex-seeking GSN apps to search for potential sex partners. Most men in the study had multiple active accounts on sex-seeking GSN apps and allowed these apps to run much of the day. These findings are consistent with Western MSM use of GSN apps to connect with potential sex partners [12].

That Indian MSM have multiple profiles potentially allowing them to present themselves as someone who prefers to be insertive, receptive, or versatile and someone who does or does not endorse condom use during sex complicates how we understand the presentation of self on GSN apps. That MSM in the Greater Mumbai or Thane region endorsed a preference for both using condoms for anal sex and engaging in condomless anal sex further complicates how we understand the way in which Indian MSM present themselves in an online space. Regarding the use of multiple profiles, Ross and colleagues [3,18] and other researchers [5] suggest that MSM, who are often stigmatized due to a lack of acceptance of their same-sex attraction, use online sex-seek spaces to experiment with sexual identities. In India where homosexual behavior is stigmatized and same-sex behavior is illegal [19], the use of multiple profiles with different sexual preferences could allow men the freedom to virtually experiment and fantasize about engaging in various sexual behaviors, which could aid in sexual identity development and connectedness to other MSM. Distinguishing between profiles they use to meet sex partners offline versus meeting partners to engage in chatting or virtual sex—which would allow for the enactment of sexual behaviors without placing oneself at risk of HIV or STI transmission—was beyond the scope of this study. Few MSM in our sample disclosed their HIV or STI status on their profiles. This result could be explained in the context of the stigmatized culture that Indian MSM encounter or that a majority of the sample did not know their HIV or STI status. We believe it is important to consider the low levels of HIV disclosure on GSN apps as an important behavior to understand, as we know very little about the online Indian MSM community. Western literature suggests that online spaces are typically anonymous and offer an opportunity to create a virtual identity that may not match the nonvirtual identity. Therefore, it is important to explore how virtual identities impact HIV disclosure for Indian MSM. If in future research we find that the use of some profiles contributes to risk while others are protective, there would be an opportunity to educate MSM about how to use the profiles created on GSN apps to fulfill their sexual desires while minimizing risk.

Men living inside the Greater Mumbai or Thane region were more likely to endorse using condoms than MSM living outside the MSA. The difference between groups in stated condom preference could be the result of HIV prevention services and outreach being concentrated within the physical cruising sites of Greater Mumbai or Thane region, suggesting a possible overlap between populations on physical and virtual cruising platforms. HIV prevention services should now also be targeted toward encouraging Internet-using MSM to disclose their condom use preferences on GSN apps to facilitate conversations between potential sex partners about condom use before meeting for a sexual encounter.

### Limitations

The results of this study should be considered within the limitations of this understudied population. Due to the rapid changes in technology and rapid creation of mobile apps, the GSN apps reported in this paper may increase or decrease in use overtime. On average, the participants were highly educated, employed, and self-identified as either bisexual or gay, but were not out or open about their sexual identity to most people in their lives. Because the study was cross-sectional, we could not determine if disclosing a preference for condomless anal sex on GSN apps leads to an increased risk for HIV or STIs among Indian MSM or vice versa. There is robust evidence supporting the connection between condomless anal sex and increased risk for HIV transmission [20]. However, there is no evidence that we are aware of, that confirms a connection between stating a preference for condomless anal sex and risk for HIV transmission within online venues among Indian MSM populations. Furthermore, stated preferences for condom use were based on self-reports, which might explain the discrepancy between high reporting for both protected and condomless anal sex. The discrepancy in self-reported condom preference on GSN apps could be explained by participants having multiple profiles to endorse their current condom-use desires. Therefore, more research is needed to understand how Indian MSM report condom preferences on GSN apps and its connection to actual condom-use.

### Conclusions

The purpose of this study was to describe how Indian MSM present themselves on GSN apps, how their presentation contributes to sexual preferences, and if the presentation differs due to proximity to the Greater Mumbai or Thane region. Our data begins to fill the gap in understanding these aspects. As mobile penetration in India continues to grow and smartphone use increases, the use of GSN sex-seeking apps by MSM should also increase, potentially increasing the risk of HIV or STI transmission within the app’s closed sexual networks. Considering that HIV interventions are solely focused on physical cruising sites in India, our findings highlight the need for Indian HIV Interventions to look beyond current scopes and expand the ambit of HIV interventions to virtual platforms as well. Interested researchers, including mobile interventions, should continue to monitor how MSM use mobile technology to meet male sex partners.

## Abbreviations

GSN: geosocial networking
HIV: human immunodeficiency virus
LGBT: lesbian, gay, bisexual, transgender
MSM: men who have sex with men
STI: sexually transmitted infection
